# Biofunctionalization of silk fibroin scaffolds with enamel matrix protein and injectable platelet rich fibrin for soft tissue augmentation: an *in-ovo* study

**DOI:** 10.1186/s40729-025-00601-1

**Published:** 2025-02-20

**Authors:** Diana Heimes, Nadine Wiesmann-Imilowski, Timpe Heidebrecht, Sebastian Blatt, Andreas Pabst, Philipp Becker, Sandra Fuest, Jürgen Brieger, Ralf Smeets, Peer W. Kämmerer

**Affiliations:** 1 Department of Oral and Maxillofacial Surgery, University Medical Center Mainz, Augustusplatz 2, 55131 Mainz, Germany; 2 Department of Oral and Maxillofacial Surgery, Federal Armed Forces Hospital, Rübenacherstr. 170, 56072 Koblenz, Germany; 3 Department of Oral and Maxillofacial Surgery, University Medical Center Hamburg- Eppendorf, Martinistraße 52, 20246 Hamburg, Germany; 4 Department of Otorhinolaryngology, University Medical Center Mainz, Langenbeckstrasse 1, 55131 Mainz, Germany

**Keywords:** Enamel matrix protein, Emdogain^®^, Injectable platelet-rich fibrin, iPRF, Silk fibroin, Chorioallantoic membrane assay, CAM assay, Biofunctionalization, Tissue engineering

## Abstract

**Purpose:**

Silk fibroin (SF) is a biomaterial derived from the cocoon of the mulberry silkworm. This study aimed to assess the capacity of SF matrices biologized with injectable platelet-rich fibrin (iPRF) or enamel matrix protein (EMP) to modulate angiogenesis and immune response in the chorioallantoic membrane (CAM) assay.

**Methods:**

300 eggs were divided into the following groups: CM + NaCl, CM + iPRF, CM + EMP, SF + NaCl, SF + iPRF, and SF + EMP. Matrices were applied to the CAM on embryonic development day (EDD) 7 after rehydration. Angiogenesis, represented by vascularized area, vessel density, and vessel junctions, was evaluated on EDD 10, 12, and 14. Additionally, gene expression of HIF-1ɑ, VEGF, MMP-13, and NOS2 was assessed via quantitative polymerase chain reaction (qPCR) on EDD 11 and 14.

**Results:**

The number of vascularized specimens was notably higher in SF matrices regardless of the treatment applied, while in the CM group, only matrices biofunctionalized with iPRF demonstrated vascularization. On EDD 14, the CM + iPRF group exhibited the highest values for total vascularized area (CM + iPRF: 57.52%, SF + iPRF: 21.87%, *p* < 0.001), vessel density (CM + iPRF: 0.0067 μm/µm^2^, SF + iPRF: 0.0032 μm/µm^2^, *p* = 0.002), number of vessel junctions (CM + iPRF: 14.45, SF + iPRF: 4.82, *p* = 0.001). Gene expressions displayed high data variability and no significant differences between the groups.

**Conclusions:**

Biofunctionalization with iPRF in CM leads to a high vascularization rate probably through their capability of retaining higher liquid volumes, suggesting improved intraoral wound healing after guided tissue regeneration (GTR). Despite biofunctionalization, SF matrices exhibit a high vascularization, indicating SF as a promising material for GTR.

## Introduction

In oral and maxillofacial surgery, various transplant materials of different origins replace deficient or defective tissues. Guided tissue regeneration (GTR) aims to form and restore oral soft tissues. The replacement materials are categorized into preparations from autologous, allogeneic, xenogeneic, and alloplastic origin [1].

Carrier systems in tissue engineering are based on scaffolds that serve as a matrix for cellular invasion and integration, facilitating the ingrowth of blood vessels and thus enabling the integration of foreign material into a defect, and soft tissue regeneration [2]. Extracellular matrix components such as collagen, fibronectin, elastin, and polymers have often been used as “natural” substitute materials [3, 4]. Still, despite their promising results, they have crucial disadvantages. Among these are poor mechanical resilience and limited reproducibility of the material composition. This contrasts with synthetic polymers, such as polyurethane and polycaprolactone, which have good mechanical properties but can also lead to an increased adverse immune response and delayed regeneration due to the release of acidic degradation products [5, 6].

Although autologous grafts are currently considered the clinical gold standard, particularly for use as mucosal substitutes or soft tissue thickening, an increasing number of pre-diseased patients with risk factors for adverse events associated with autologous tissue harvesting necessitates effective alternatives [7]. Using xenogeneic, allogeneic, or alloplastic matrices reduces operative time and postoperative morbidity, notably decreasing pain, swelling, and bleeding [8–10]. Especially in light of demographic trends, soft tissue matrices are gaining importance in implantology. Silk fibroin (SF) matrices are becoming increasingly relevant due to their biocompatibility [11] and mechanical strength [12] providing favorable conditions to meet the high demands across various indications.

Three-dimensional printing is considered a crucial technology in material engineering for biomedical applications and is becoming increasingly important in fabricating patient-specific grafts. The usability of SF for the fabrication of complex three-dimensional scaffolds has been demonstrated several times, an important prerequisite for individualized tissue substitutes via tissue engineering [2].

Silk, produced by the silkworm, is a product that has been used for textile applications for more than 4,000 years and is known for its outstanding mechanical properties. The FDA first approved silk as a suture material in 1987, and it has since become the standard of care [12], so silk fibroin is more of a renaissance than a novelty. SF is an absorbable biomaterial isolated from the cocoon of the mulberry silkworm *Bombyx mori*. Using a gentle dissolution process, SF can be converted into an aqueous suspension and then processed in a highly versatile manner into different forms and structures such as scaffolds, membranes, fibers, hydrogels, etc [11]. A unique feature here is the combination of adjustable biological and mechanical properties (e.g., degradation rate and stiffness) while maintaining high biocompatibility [13–16]. The ability to fabricate 3D-printed, patient-specific structures that faithfully reproduce the morphology of physiological tissue makes silk fibroin products a preferred biomaterial for tissue engineering applications [14].

Modifying the applied matrices to increase the proangiogenic effect is desirable to improve the already observed promising results in vitro and in vivo. Biofunctionalization aims at imparting specific biological properties to carrier materials through the targeted addition of active substances. Given the already proven proliferation-promoting and pro-angiogenic properties of the endogenous blood product Platelet-rich fibrin (PRF) and the commercially available enamel matrix protein (EMP), which recently underwent an expansion of the indication spectrum due to convincing data, these two substances were selected for biofunctionalization of the SF scaffolds to be tested.

PRF is a frequently used agent in many indications. This platelet- and growth factor-rich matrix can be obtained in solid or liquid form from venous blood by centrifugation. Numerous studies demonstrated positive effects of PRF on cell differentiation, migration, and proliferation ability [17–23]. Thus, its pro-angiogenic properties represent an ideal candidate for biofunctionalization in tissue engineering [24–26]. Advantages also include the ease of production, simplicity of handling, and minimal cost of a product manufactured bedside from the patient’s blood [21].

Initially developed for the regeneration of periodontal defects [27, 28], EMP exhibits similar proliferation-promoting properties when applied to collagen matrices [29]. However, in vivo experiments show heterogeneous results in healing oral mucosal defects by both PRF and EMP, directly comparing the angiogenic and immunomodulatory properties of both compounds in the biofunctionalization of different matrices attractive [30]. EMP positively affects cell viability, proliferation, and differentiation. In addition to its impact on angiogenesis and soft tissue regeneration, EMP enhances the transcription of various growth factors and cytokines [27, 31]. Animal studies showed a positive effect on healing acute and chronic wounds [28, 31].

SF matrices have mainly been used as wound dressings to improve dermal healing and treat liver, vascular, and nerve damage; dentistry focuses on bony regeneration [12, 16]. This study is the first to investigate neovascularization and immune response to biofunctionalized SF matrices *in ovo*. Little data has been available, especially regarding the immunogenicity of the material in the living organism. Especially the combination with proliferation-promoting substances such as PRF and EMP has not been investigated so far and thus is of particular interest. Therefore, this *in-ovo* study aimed to assess vascularization and immune response in SF matrices compared to CM, both biologized with either iPRF or EMP, in the chorioallantoic membrane (CAM) assay.

## Materials and methods

### Study design

On egg development day (EDD) 7, either a SF scaffold (experimental group) or a collagen matrix (control group) was placed onto the chorioallantoic membrane (CAM) of the developing chicken embryo. Before application, the membranes were either biofunctionalized with (I) enamel matrix protein (EMP), (II) injectable platelet-rich fibrin (iPRF), or set as control (rehydrated with 0.9% saline solution; NaCl). In total, 300 eggs were analyzed. Intravital fluorescent microscopy (EDD 10, 12, 14) and quantitative polymerase chain reaction (qPCR; EDD 11, 14) were used to determine vascularization and neoangiogenesis. One-third of the embryos were euthanized on EDD 11 and the rest at EDD 14 for qPCR or (immuno-)histochemical staining.

### Matrices

#### Production of silk solution, PureSilk^®^

The SF aqueous solution was obtained using PureSilk^®^ technology (Fibrothelium GmbH, Aachen, Germany), which enables to provide medical-grade quality on an industrial scale for a broad range of concentrations. Briefly, fibroin was separated from sericin by degumming it in a hot alkali solution before dissolving it in a proprietary, non-toxic solvent system based on Ajisawa’s reagent. The dissolved fibroin was fully dialyzed against VE water within 8 h using tailored extraction processing. The fibroin concentration used in this study was adjusted to 3 wt% and stored at 4 °C.

#### Production of electrospun non-woven

SF solution (PureSilk^®^) was used to prepare the electrospun non-woven. A needleless electrospinning set-up was used, based on a spinneret and a drum collector. The voltage applied was between 25 and 30 kV using a distance of 30 cm. The spinning speed was 100 rpm, and the spinning time was one hour. Spinning was performed at room temperature and 30–45 rh relative humidity. Subsequent crosslinking of the material was unnecessary due to a prior solution modification. Samples were finally punched to a diameter of 12 mm (punching iron) and stored in suitable pouches.

#### Collagen matrix

The collagen matrix Mucoderm^®^ (botiss biomaterials GmbH, Zossen, Germany) is a porcine-derived, three-dimensional, native collagen matrix composed of collagen type I and III without further chemical treatment. It is commercially available with a 1.2–1.7 mm thickness and dimensions of 15 × 20 mm, 20 × 30 mm, or 30 × 40 mm. According to the manufacturer, it is intended to be used as a soft tissue graft. Its porous collagen network is meant to act as a guiding structure for cell migration. In oral and maxillofacial surgery, it is used for soft tissue augmentation, the closing of extraction sockets, and the thickening of peri-implant tissue [32].

### Enamel matrix protein

The commercially available product Emdogain^®^ (Straumann Group, Basel, Switzerland) contains EMPs derived from embryonic porcine tooth germs in a propylene glycol alginate carrier substance. Before usage, the gel was diluted with 0.9% NaCl (Braun Melsungen AG, Melsungen, Germany) to a concentration of 100 µg/ml.

### Injectable platelet-rich fibrin (iPRF)

Venous whole blood was taken from one examiner (DH) upon written consent using a butterfly needle and a 10 ml S-PRF tube (mectron Deutschland Vertriebs GmbH, Cologne, Germany). Centrifugation was performed at 12 × 100 rpm for 7 min at room temperature using a PRF centrifuge with a relative centrifugal force 177 g at a fixed angle rotor with a radius of 110 mm (PRF Duo quattro Centrifuge, mectron Deutschland Vertriebs GmbH, Cologne, Germany). All procedures were conducted in agreement withthe Declaration of Helsinki and approved by the Ethics Committee of Landesärztekammer Rhineland-Palatine (no. 2019–14705_1).

### Biofunctionalization

**Fig. 1:**
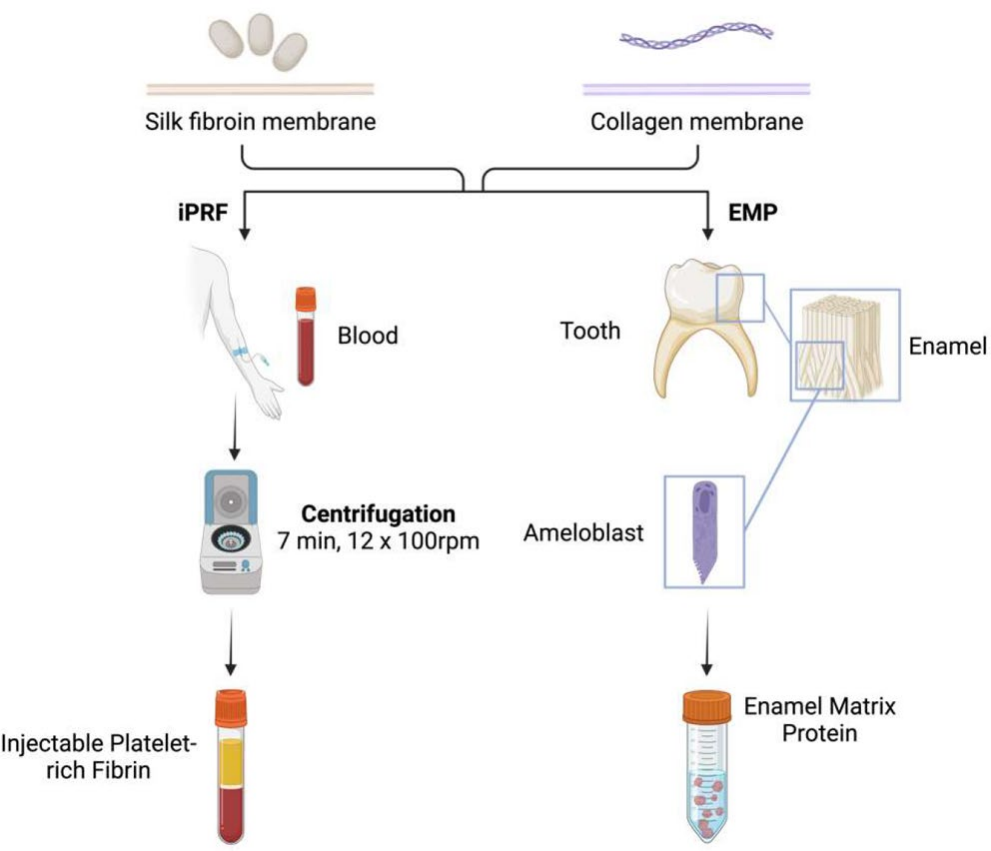
Biofunctionalization. Patient’s own blood is used for the production of iPRF and processed through centrifugation. The fibrin present in the uppermost phase was utilized in the biofunctionalization of the matrices. EMP is produced by ameloblasts in the tooth germ and was acquired as a commercial material for this experiment. The ready-to-use mass was applied in diluted form onto the silk fibroin and collagen matrices. Abbreviations: iPRF = injectable platelet-rich fibrin, EMP = enamel matrix protein. Created with BioRender.com

The matrices were biofunctionalized using 400 µl of solution (either NaCl, iPRF, or EMP) for rehydration over 10 min. Before application to the CAM, excess fluid was then eliminated by placing the matrices on sterile filter paper (Tissue-TEK II Filterblock, Vogel GmbH & Co.KG, Giessen, Germany) for 10 s (Fig. 1).

### Quantification of absorbed liquid mass per matrix

To assess the amount of liquid absorbed by the matrices during biofunctionalization, the respective matrices were weighed, soaked in 400 µl of NaCl for rehydration over 10 min, and then weighed again.

### *In Ovo* Chorioallantoic membrane assay (CAM Assay)

Fertilized white Leghorn chicken eggs (LSL Rhein-Main GmbH, Dieburg, Germany) were incubated at 37.5 °C with constant humidity (50%) in an incubator (Type 3000 digital and fully automatic, Siepmann GmbH, Herdecke, Germany) and further processed as described by Heimes et al. [33]. Summed up, eggs were placed horizontally on one side for three days to ensure detachment of the CAM from the eggshell. On EDD 3, 5–6 mL of the albumin was removed to enlarge the space between the eggshell and CAM. Then, a small 2 × 3 cm window was cut into the upwards-pointing part of the eggshell and covered with Parafilm^®^ (Sigma-Aldrich, St. Louis, MO, USA) to prevent evaporation.

**Fig. 2:**
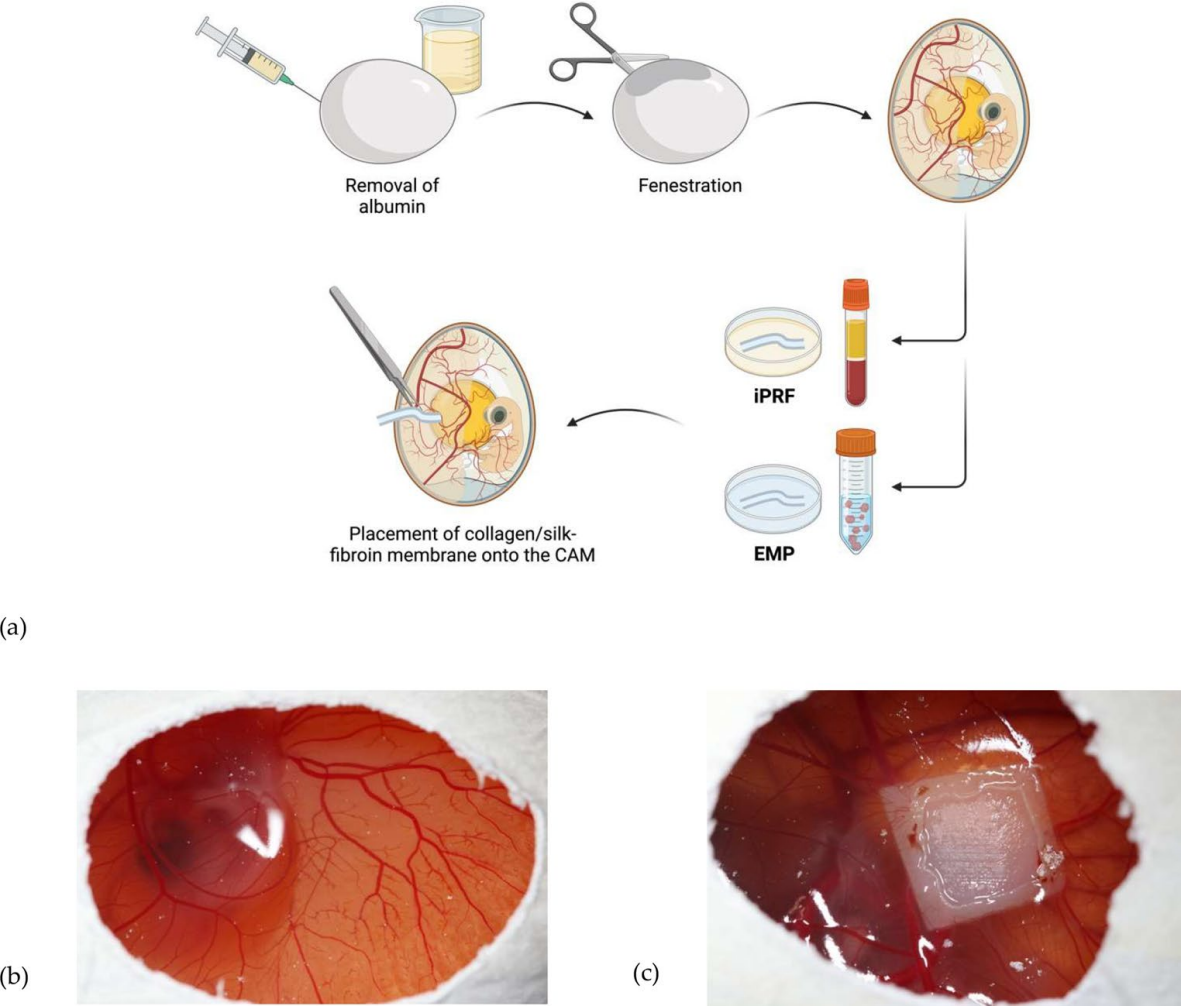
(**a**) Workflow. Removal of a few milliliters of albumin causes a lowering of the egg white and enables the fenestration of the eggshell on EDD 3. After the matrices were biofunctionalized with iPRF and EMP, they were placed on the CAM on EDD 7. (**b**) Seven-day-old chicken embryo. After cutting a small window in the upper part of the eggshell, the CAM is accessible for matrix application. (**c**) The ingrowth and overgrowth of soft tissue and vessels are clearly visible through the window. Abbreviations: CAM = chorion allantois membrane, EDD = egg development day, iPRF = injectable platelet-rich fibrin, EMP = enamel matrix protein. (**a**) Created with BioRender.com

On EDD 7, matrices were cut into pieces of 25 mm^2^, biofunctionalized as described, and placed onto the CAM (Fig. 2).

**Fig. 3:**
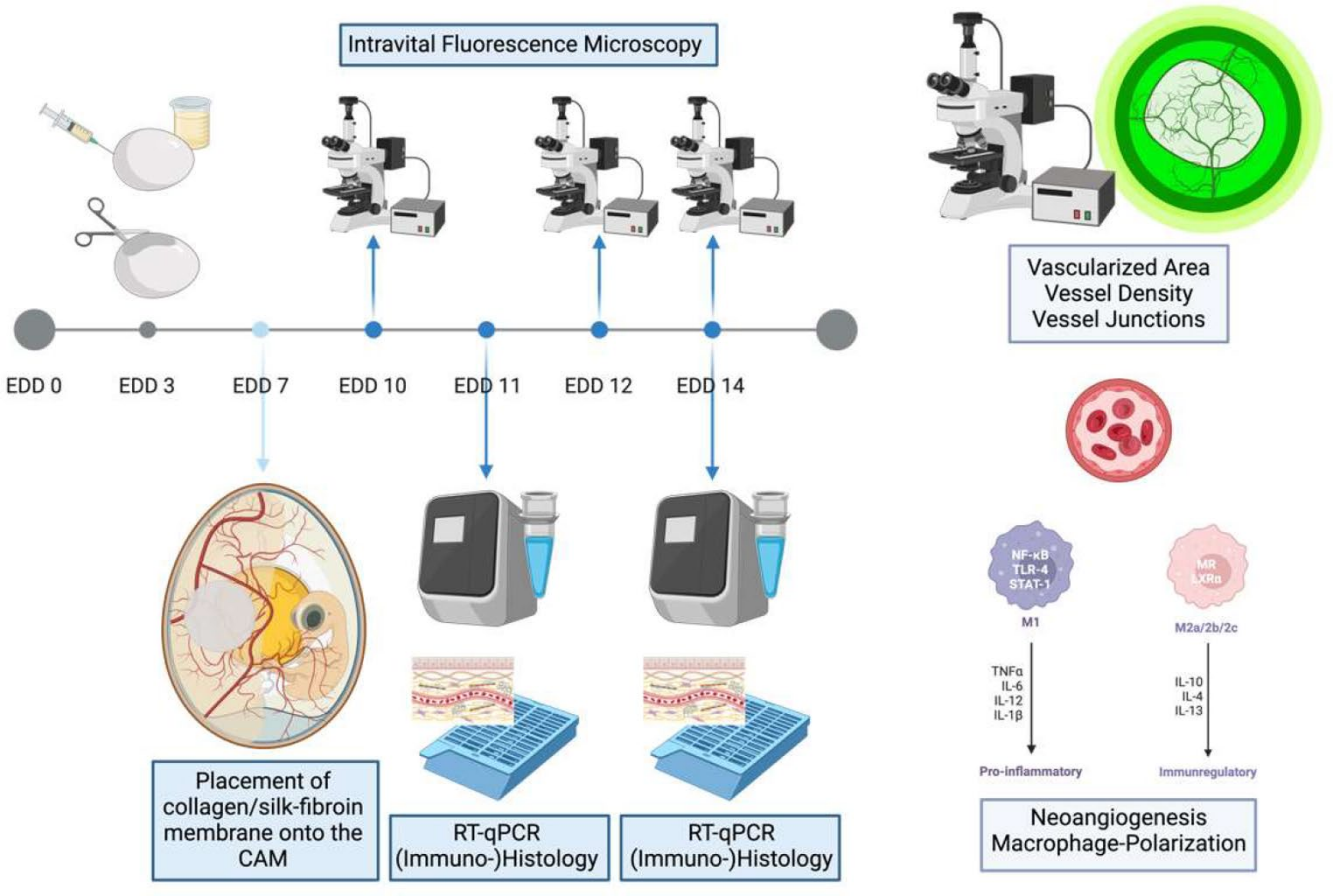
Experimental procedure in schematic overview. Following the opening of the eggshell on EDD 3 and the placement of the matrices on the CAM (EDD 7), the analysis of neovascularization was conducted using intravital fluorescence microscopy on EDD 10, 12, and 14. Samples for mRNA analysis using RT-qPCR and histological staining were collected on EDD 11 and 14. Abbreviations: EDD = egg development day, CAM = chorion allantois membrane, RT-qPCR = quantitative reverse transcription polymerase chain reaction. Created with BioRender.com

After three days, pictures were taken with a digital intravital fluorescence microscope (Olympus BXFM, Olympus Deutschland GmbH, Hamburg, Germany) at a 50-fold magnification using the cellSens Dimension software package. One-third of the eggs per group were euthanized on EDD 11 to assess the early tissue response by mRNA analysis via qPCR and histochemistry. The remaining embryos were further incubated, and vascularization was analyzed again using the digital intravital fluorescence microscope at EDD 12 and EDD 14. After that, the remaining embryos were euthanized on day 14 by cutting the main vessels. A sample of the matrices and the surrounding CAM was extracted for qPCR or histochemical staining (Fig. 3).

### Intravital fluorescence microscopy

Intravital fluorescence microscopy was performed using an in vivo microscope (Olympus BXFM, Olympus Deutschland GmbH, Hamburg, Germany) at a 50-fold magnification and the cellSens Dimension software package by Olympus.

### Determination of the vascularized area

Intravital fluorescence microscopy was performed at EDD 10, 12, and 14. Multiple image arrays were generated using the software package’s automated composing function. After that, pictures were pseudonymized, and the same examiner performed all measurements. Determining the vascularized area was done according to Heimes et al. [33], first marking the matrix’s outer border and then the inner border of the vascularized area. The software automatically calculated the marked areas. To receive the size of the vascularized area, the non-vascularized area in the center of the matrix was subtracted from the site of the whole matrix. The proportion of the vascularized area per matrix and the increase in vascularized area over time were calculated.

### Vessel density and vessel junction analysis

Measurements were performed at EDD 10, 12, and 14 to determine vessel density and junction analysis. The focus of the intravital fluorescence microscope was set at two pre-determined regions of interest (one in each corner of the matrix), and pictures were taken. After that, pictures were pseudonymized, and the same examiner performed all measurements. Using the software package cellSens Dimension, a 1000 × 1000 μm grid was laid over the image. All vessels visible inside the ROI were marked manually. The software automatically calculated the overall length of all vessels marked inside the ROI and the vessel density per µm^2^ calculated by division of the overall vessel length by the marked area of 1,000,000 µm^2^. The vessel density of both ROIs was summed. The underlying equation is described elsewhere [33]. Additionally, the increase in vessel density over time was calculated.

Vessel junction analysis was performed using the generated dataset edited using the open-source software Fiji, a distribution of ImageJ. The number of branches and junctions per image was automatically calculated. The number of vessel junctions of both ROIs was summed, and the increase in vessel junctions over time was calculated.

### mRNA isolation and quantitative polymerase chain reaction (qPCR)

To isolate the total cellular mRNA, parts of the CAM being in contact with the matrix (one probe per opposite edge of the matrix) were cut out at EDD 11 and EDD 14 and immediately transferred to cryotubes and shock-frozen in liquid nitrogen. Digestion was performed with the homogenizer Speedmill Plus (Analytik Jena AG, Jena, Germany) using innuSPEED Lysis Tubes E (Analytik Jena AG, Jena, Germany) according to the manufacturer’s specifications. The following RNA isolation was performed with the RNeasy Mini Kit (Qiagen N.V., Venlo, Netherlands) according to the manufacturer’s specifications, including a DNase digestion. The concentration and purity of the obtained RNA were determined with the NanoDrop™ One (Waltham, MA, USA). RNA transcription to cDNA was done with the iScriptTM cDNA Synthesis Kit 100 (Bio-Rad Laboratories Inc., Hercules, CA, USA), and obtained cDNAs were stored at − 20 °C. SYBR green-based qPCRs were performed in triplicate, including 1 µL cDNA in each PCR reaction using the iTaq Universal SYBR Green Supermix (Bio-Rad Laboratories Inc., Hercules, CA, USA) with the CFX Connect Real-Time PCR Detection System (Bio-Rad Laboratories Inc., Hercules, CA, USA). The Ribosomal Protein L4 (RPL4) gene was used as an endogenous reference. Its suitability for normalization was determined with the Normfinder Excel Add-in (https://moma.dk/normfinder-software). The transcripts were quantified by the ∆∆CT method using an internal calibrator sample, which was included in all qPCR runs. Gene expression levels are shown in relative on to the internal calibrator. qPCR analyses were performed for VEGF, HIF-1ɑ, MMP-13, and NOS2. Data analysis was performed using Bio-Rad CFX Manager software 3.1 (Bio-Rad Laboratories Inc., Hercules, CA, USA).

### Histochemical staining

HE staining was performed by removing the paraffin from the slide. Afterward, slides were incubated in an isopropanol solution with decreasing concentrations (100%/100%/96%/70%/50%) for 5 min. Subsequently, each slide was washed in purified water (20 min.) and incubated in Mayer’s hemalum solution (Carl Roth GmbH + Co. KG, Karlsruhe, Germany) for 5 min. The slides were washed using purified water (10 min.) and incubated in eosin (Merck KGaA, Darmstadt, Germany) for 1.5 min. The slides were again washed (10 s.) and incubated in isopropanol solution with increasing concentrations (70%/96%/100%) and cleared using ROTI^®^Histol (Carl Roth GmbH + Co. KG, Karlsruhe, Germany) for 10 min. Finally, specimens were embedded in Eukitt^®^ (Sigma-Aldrich, St. Louis, MO, USA) for microscopy.

### Statistics

The primary outcome parameter was angiogenesis, analyzed by fluorescence microscopy which was represented by the vascularized area, the vessel density, and the vessel junctions.

As a secondary outcome parameter, gene expression of HIF-1ɑ, VEGF, MMP-13, and NOS2 was assessed via quantitative polymerase chain reaction.

Due to the exploratory nature of the study and the small sample size, which prevented a sufficient test for normal distribution, such a test was not carried out. Data was analyzed using GraphPad Prism Software (Version 6.01, GraphPad Software, La Jolla, CA, USA). Values were presented as mean ± standard deviation (SD) unless stated otherwise. Differences between the groups over time were analyzed using two-way ANOVA, followed by post hoc Bonferroni corrections for pairwise comparisons. A p-value < 0.05 was considered statistically significant (* *p* < 0.05, ** *p* < 0.01, *** *p* < 0.001, **** *p* < 0.0001). Data was obtained from five independent experimental runs. In the following, n refers to the number of eggs used per experimental group.

## Results

### Quantification of absorbed liquid mass per matrix

The collagen matrices absorbed an average of 19.64±5.91 µL of NaCl (density: 1.005 g/cm³), whereas the amount of liquid absorbed by SF-matrices was considerably lower at 0.74±0.44 µL (*n* = 5 each; *p* < 0.001).

### Proportion of vascularized matrices

**Fig. 4:**
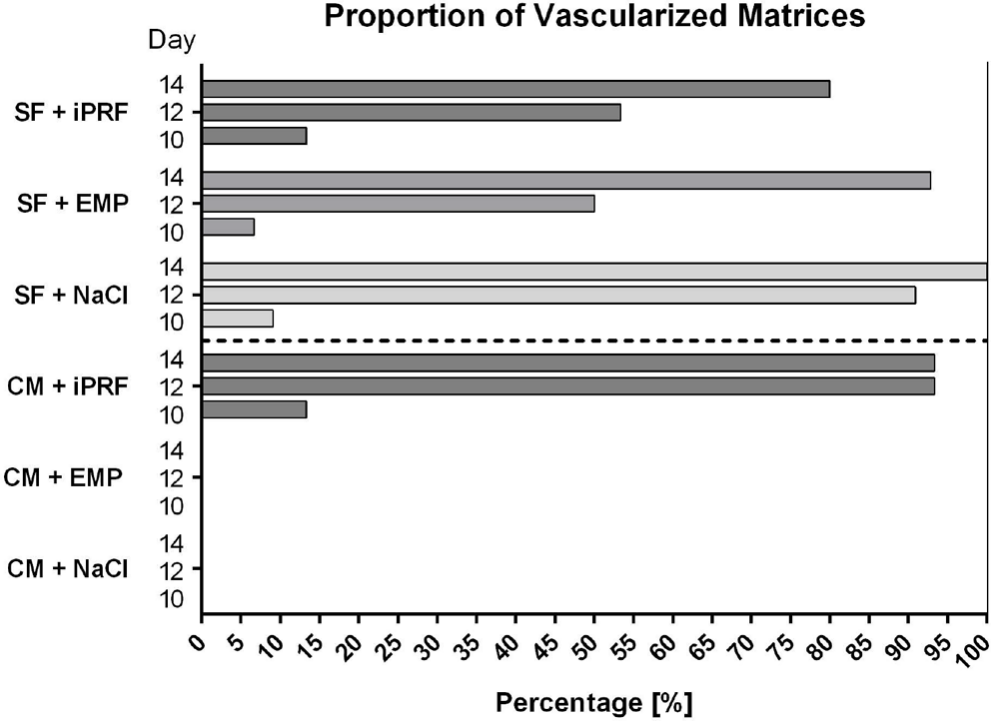
Percentage of vascularized matrices on EDD 10, EDD 12 and EDD 14. The percentage of vascularized matrices relative to the total number of matrices in the experimental groups is depicted as a bar chart. The data was obtained from five independent experimental runs. Number of matrices assessed: CM + NaCl *n* = 9, CM + iPRF *n* = 15, CM + EMP *n* = 17, SF + NaCl *n* = 11, SF + iPRF *n* = 15, SF + EMP *n* = 14. Abbreviations: CM = collagen matrix, SF = silk fibroin matrix, NaCl = sodium chloride, PRF = platelet-rich fibrin, EMP = enamel matrix proteins

Substantial differences were observed upon analyzing the vascularization proportions of matrices from both experimental groups. Three days (EDD 10) after placement, only 2/15 silk fibroin matrices and 2/15 collagen matrices rehydrated with iPRF, 1/14 SF matrix biofunctionalized with EMP and one SF matrix from the control group did show signs of vascularization. However, on EDD 12, intriguingly, the control group (CM + NaCl) did not show any vascular structures either on or within the matrix, whereas 93.33% of matrices in the CM + iPRF group were vascularized. CM matrices treated with EMP demonstrated vascularization in 0% of cases. In contrast, SF rehydrated with NaCl matrices were vascularized in 90.9%, those treated with EMP in 50% of cases, and the iPRF group demonstrated vascularization in 53.33% of cases (Table 1; Fig. 4).

**Table 1:** Proportion of vascularized matrices on EDD 12

Group	CM + NaCl	CM + iPRF	CM + EMP	SF + NaCl	SF + iPRF	SF + EMP
Vascularized (n)	0/9	14/15	0/17	10/11	8/15	7/14
Percentage (%)	0	93.33	0	90.9	53.33	50

Two days later, on EDD 14, CM rehydrated with NaCl, and those treated with EMP still did not display any vascularization. However, CM biofunctionalized with iPRF showed vessel ingrowth in 93.33% of cases. SF matrices rehydrated with NaCl exhibited vascularization in 100% of cases, while the iPRF group showed vascularization in 92.86% of cases and the EMP group in 80% of cases (Table 2; Fig. 4).

**Table 2:** Number of vascularized matrices on EDD 14

Group	CM + NaCl	CM + iPRF	CM + EMP	SF + NaCl	SF + iPRF	SF + EMP
Vascularized (n)	0/9	14/15	0/17	11/11	12/15	13/14
Percentage (%)	0	93.33	0	100	80	92.86

### Proportion of vascularized surface area

In all experimental groups, a progressive increase in the proportion of vascularized surface area was observed throughout the experiment. In most instances, the amount of vascularization doubled between EDD 12 and EDD 14. On EDD 12, the CM + iPRF group showed 26.59 ± 5.01% vascularization of the surface (*n* = 15). In contrast no assessment of vascularization was possible for CM treated with EMP (*n* = 16) and NaCl (*n* = 9) due to a lack of adherence to the CAM. For SF matrices, rehydration with NaCl resulted in 9.94 ± 8.08% vascularized surface area (*n* = 11), iPRF treatment led to 13.26 ± 10.65% (*n* = 15), and EMP biofunctionalization resulted in 10.47 ± 15.35% (*n* = 14) on EDD 12. Due to the lacking vascularization of CM rehydrated with NaCl and EMP, statistically significant higher values were observed in comparison to CM biofunctionalized with iPRF (*p* < 0.005).

**Fig. 5:**
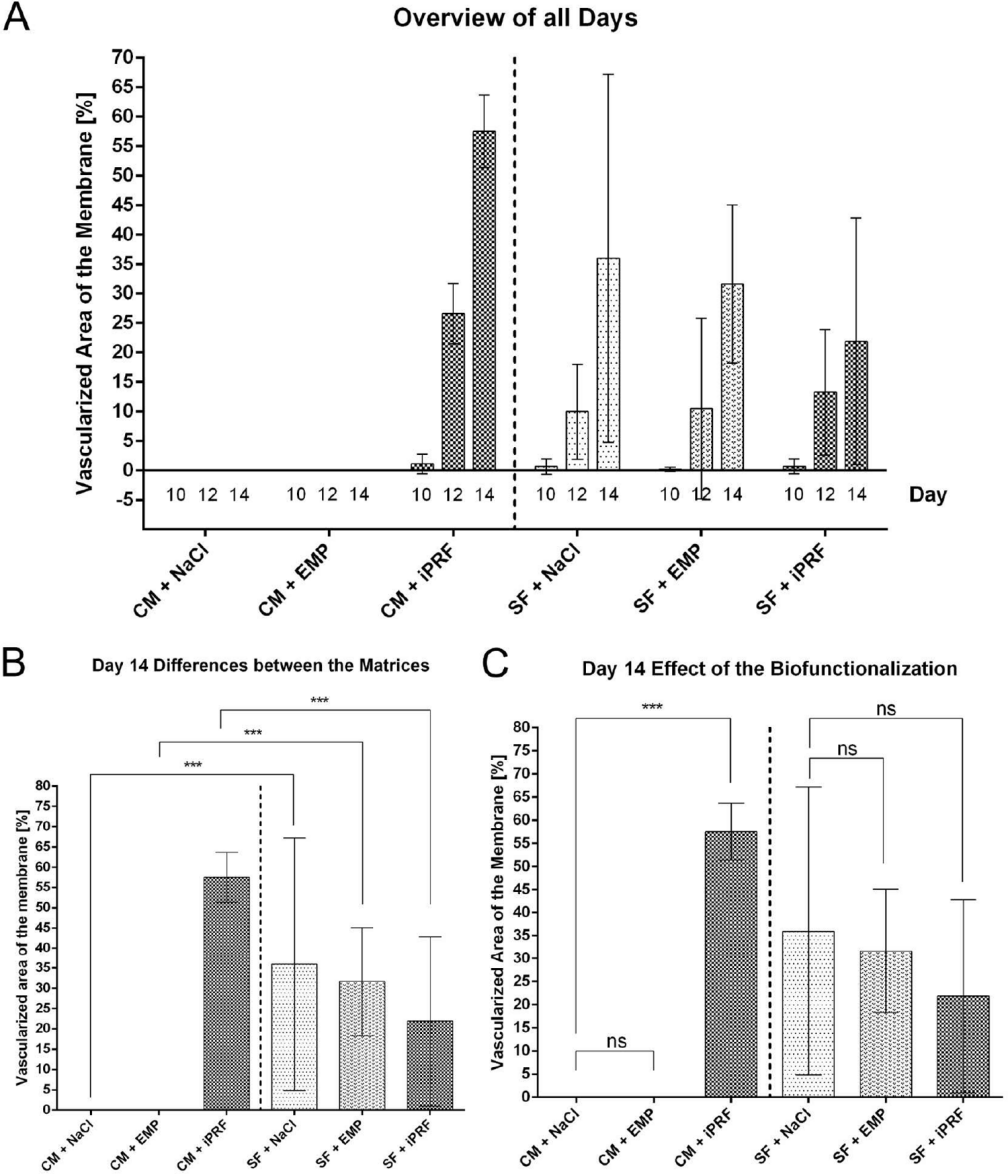
(**A**) Proportion of vascularized surface area of the matrix on EDD 10, 12 and 14. The percentage of vascularized surface area relative to the total matrix surface was depicted as bar charts. There was a strong increase in the vascularized area from EDD 10 to EDD 12 and a doubling from EDD 12 to EDD14 in CM + iPRF and all SF groups independent of the type of rehydration. In contrast CM rehydrated with NaCl or EMP did not show any vascularization of the matrix throughout all days under investigation (**B**) The graph depicts the differences between the groups focusing on the type of matrix on EDD 14. Due to the lacking adherence of CM treated with NaCl and EMD to the CAM, SF rehydrated with NaCl or EMD showed statistically significant higher vascularization than the respective SF-matrix groups. Contrarily, CM biofunctionalized with iPRF showed the highest values on EDD 14. (**C**) The graph depicts the differences between the groups focusing on the type of biofunctionalization. CM treated with iPRF showed statistically significant higher values compared to the groups rehydrated with NaCl and EMD. All SF groups showed similar values with no significant difference between the treatment modalities. Shown are mean values ± SD. A two-way ANOVA was performed comparing between CM and SF with the same biofunctionalization to assess the differences between the matrices (**B**) and between the different biofunctionalization within each matrix group (**C**), correction for multiple comparisons with Bonferroni, (*** *p* < 0.001, ns = not significant). The data was obtained from five independent experimental runs. Abbreviations: CM = collagen matrix, SF = silk fibroin matrix, NaCl = sodium chloride, iPRF = injectable platelet-rich fibrin, EMP = enamel matrix proteins

On EDD 14, a considerably greater proportion of vascularized area was observed in all groups in comparison to EDD 12. CM treated with iPRF exhibited on average the largest vascularized area with 57.52 ± 6.16%. As with EDD 12, vascularized surface area could not be assessed for CM treated with EMP and NaCl due to a lack of detectable vessel ingrowth. In the silk fibroin control group (SF + NaCl), the vascularized area accounted for 35.98 ± 31.23%. SF treated with iPRF showed 21.87 ± 20.93%, and SF treated with EMP showed values of 31.65 ± 13.41%. Due to the lacking vascularization of CM rehydrated with NaCl and EMP, all other groups exhibited a statistically significant greater vascularized area. Furthermore, CM biofunctionalized with iPRF showed a higher vascularized area compared to SF treated with NaCl (*p* = 0.042), iPRF (*p* < 0.001) and EMP (*p* = 0.003) (Fig. 5; Table 3).

**Table 3:** Differences in the proportion of vascularized surface area of the matrices as being anlysed by a two-way ANOVA with correction for multiple comparisons by Bonferroni on EDD 14

Group	CM + NaCl	CM + iPRF	CM + EMP	SF + NaCl	SF + iPRF	SF + EMP
CM + NaCl	–	< 0.001	> 0.999	< 0.001	0.059	0.001
CM + iPRF	< 0.001	–	< 0.001	0.042	< 0.001	0.003
CM + EMP	> 0,999	< 0.001	–	< 0.001	0.037	0.001
SF + NaCl	< 0.001	0.422	< 0.001	–	0.865	> 0.999
SF + iPRF	0.059	< 0.001	0.037	0.865	–	> 0.999
SF + EMP	0.001	0.003	0.001	> 0.999	> 0.999	–

### Vessel density analysis

Like the matrices’ vascularized surface area, vessel density gradually increased throughout the experiment in those groups which exhibited vascularization. On EDD 12, CM treated with iPRF showed values of 0.00225 ± 0.0018 μm/µm² (*n* = 15). No values could be obtained for the CM + EMP and CM + NaCl group due to the absence of detectable vessel ingrowth. SF rehydrated with NaCl exhibited values of 0.0011 ± 0.0005 μm/µm² (*n* = 10), SF treated with iPRF showed values of 0.00161 ± 0.00117 μm/µm² (*n* = 13), and SF treated with EMP displayed values of 0.00057 ± 0.00087 μm/µm² (*n* = 14). No statistically significant differences were observed between the groups on EDD 12.

**Fig. 6:**
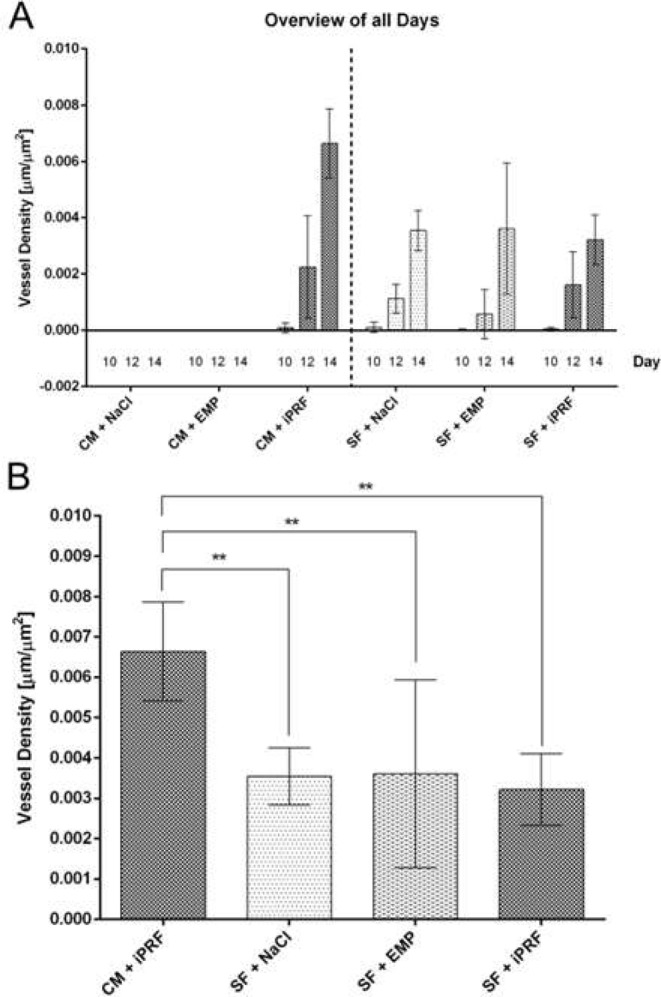
Vessel density analysis – overview of all days (**A**) and focus on EDD 14 (**B**). There was a clear increase in vessel density between EDD 10 and EDD 12 in those groups which showed vascularization. In most cases the values doubled or tripled between EDD 12 and EDD 14. On EDD 14 the number of vessels measured per µm^2^ was highest in CM treated with iPRF and differed significantly from the vessel density in all SF group. Shown are mean values ± SD. A two-way ANOVA was performed comparing between all groups, correction for multiple comparisons with Bonferroni, (** *p* < 0.01). Abbreviations: CM = collagen matrix, SF = silk fibroin matrix, NaCl = sodium chloride, iPRF = injectable platelet-rich fibrin, EMP = enamel matrix proteins

On EDD 14, CM rehydrated with iPRF exhibited values of 0.00664 ± 0.0012 μm/µm². In the SF + NaCl group, the vessel density was 0.0035 ± 0.0007 μm/µm². Vessel density in the SF + iPRF group was 0.00322 ± 0.00089 μm/µm², and in the SF + EMP group it was 0.00361 ± 0.00233 μm/µm². Significant differences were observed between CM + iPRF and SF + NaCl (*p* = 0.006) and CM + iPRF and SF + EMP (*p* = 0.002). Additionally, significantly higher vessel density was observed in CM treated with iPRF compared to the respective SF group (*p* = 0.002) (Fig. 6).

### Vessel junction analysis

The total count of vessel junctions increased over the experimental period across all groups which showed vascularization. On EDD 12, in the CM + iPRF group, the number of vessel junctions averaged 4.133 ± 5.217 (*n* = 15). The SF control group exhibited 1.083 ± 0.52 vessel junctions (*n* = 10), while the SF + iPRF group showed 1.738 ± 0.877 (*n* = 13), and the SF + EMP group had 0.35 ± 0.652 (*n* = 14). No statistically significant differences were observed among the groups.

**Fig. 7:**
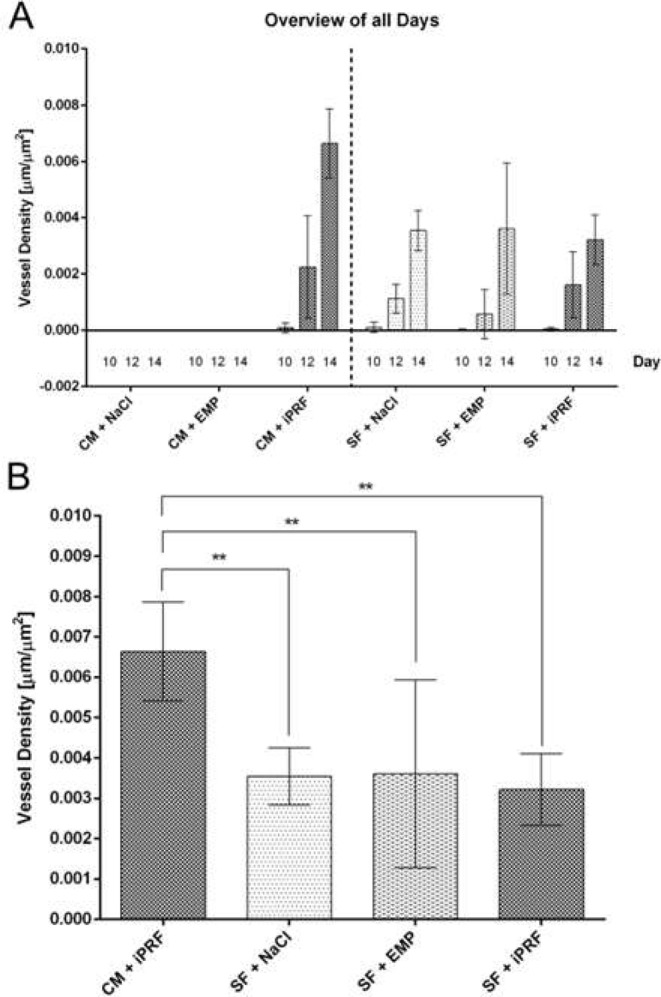
Vessel junction analysis – overview of all days (**A**) and focus on EDD 14 (**B**). There was a significant increase in vessel density between EDD 10 and EDD 12 in those groups that showed vascularization of the membrane. In most cases the values multiplied between EDD 12 and EDD 14. The total number of vessel junctions was significantly higher in CM treated with iPRF compared to the SF groups. Shown are mean values ± SD. A two-way ANOVA was performed comparing between all groups, correction for multiple comparisons with Bonferroni, (** *p* < 0.01, *** *p* < 0.001,). Abbreviations: CM = collagen matrix, SF = silk fibroin matrix, NaCl = sodium chloride, iPRF = injectable platelet-rich fibrin, EMP = enamel matrix proteins

On EDD 14, the total number of vessel junctions in CM rehydrated with iPRF was 14.445 ± 5.417. In the SF control group, there were 7.108 ± 3.809 vessel junctions, while biofunctionalization with iPRF resulted in 4.821 ± 2.144, and treatment with EMP led to 4.915 ± 2.902 vessel junctions. Significantly more vessel junctions were observed in CM treated with iPRF compared to the SF control group (*p* = 0.008), the SF group rehydrated with EMP (*p* < 0.001) and compared to the SF + iPRF group (*p* = 0.001) (Fig. 7).

### Gene expression analysis

**Fig. 8:**
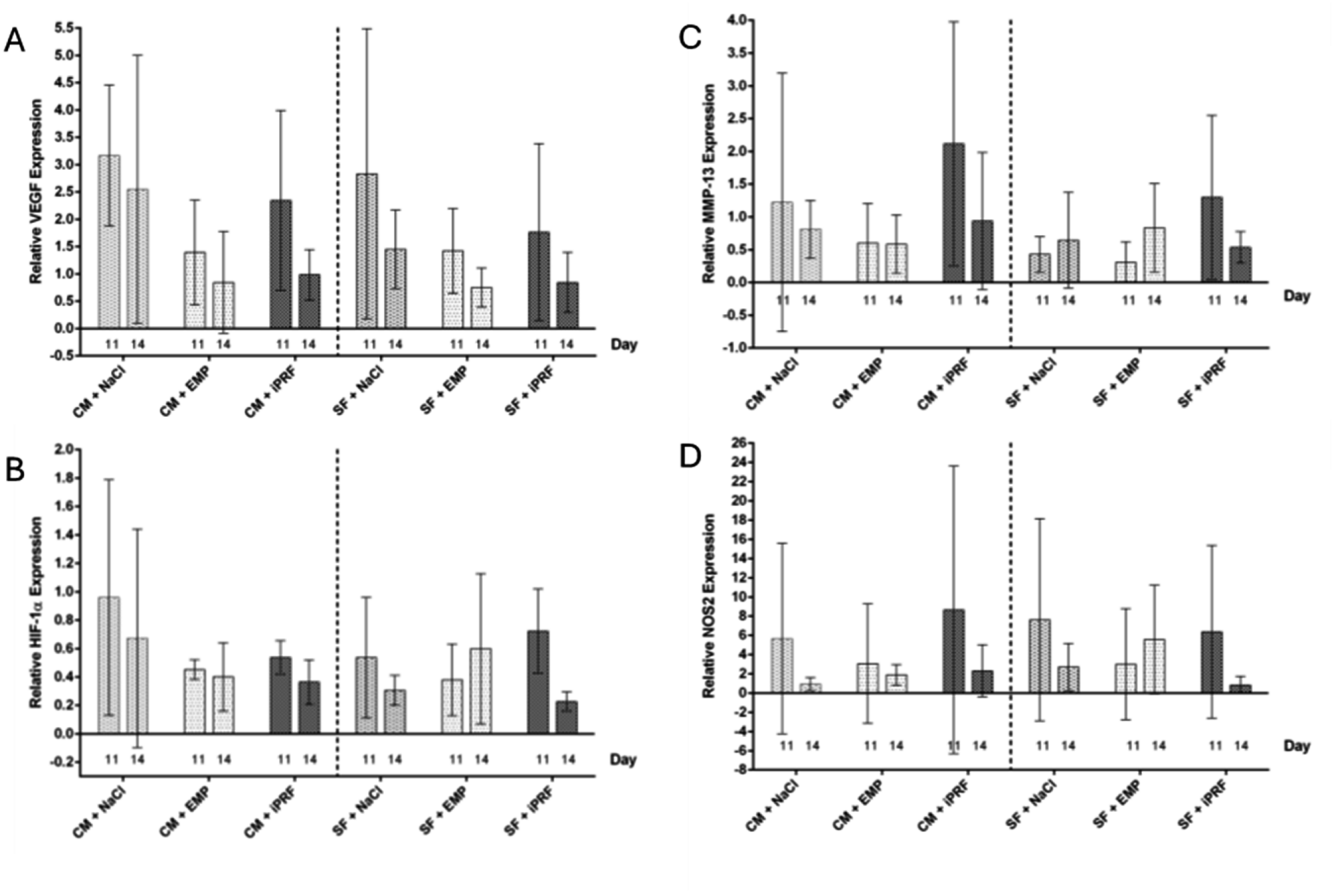
Gene expression analysis for (**A**) VEGF, (**B**) HIF-1ɑ, (**C**) MMP-13 and (**D**) NOS2 at EDD 11 and EDD 14 for all groups. (**A**) There were no statistically significant differences between the groups. VEGF expression values were higher on EDD 11 compared to EDD 14. (**B**) Similar results have been observed for HIF-1ɑ expression; here, the CM control group showed higher values than the groups biofunctionalized with either EMP or iPRF, whereas there was no such trend in the SF groups. (**C**) Contrarily, MMP-13 gene expression values were highest in the groups treated with iPRF. (**D**) NOS2 gene expressions showed a high data variability which complicated interpretation. Shown are mean values of the relative gene expression ± SD (2^-ΔΔCt values in relation to an internal calibrator). A two-way ANOVA was performed comparing between all groups, correction for multiple comparisons with Bonferroni. Abbreviations: CM = collagen matrix, SF = silk fibroin matrix, NaCl = sodium chloride, PRF = platelet-rich fibrin, EMP = enamel matrix proteins

The relative gene expression is presented in Table 4; Fig. 8. Control measurements were obtained by probing the CAM of eggs lacking any matrix (*n* = 4). Unlike the intravital fluorescence microscopy analysis, vascularized and non-vascularized specimens were examined here. As indicated in the table, no statistically significant differences were observed between the respective comparison groups (CM vs. SF), nor within the matrix groups at EDD 11 and EDD 14 for VEGF, HIF-1α, MMP-13, or NOS2.

Slight, though non-significant differences in VEGF gene expression were noted between certain groups: The CM + NaCl group exhibited higher expression levels compared to the CM group biofunctionalized with iPRF, with an even greater difference observed compared to the CM + EMP group at EDD 11. Additionally, the CM + NaCl group showed higher expression values than the untreated control group. In the intergroup comparison for SF matrices, the SF control group demonstrated higher values than the SF + iPRF group and the SF + EMP group. Although the CM group consistently exhibited the highest gene expression levels, no statistically significant differences were observed between the comparison groups (CM vs. SF) treated with iPRF and EMP.

On EDD 14, the CM + NaCl and CM + iPRF groups displayed higher VEGF gene expression levels than the CM + EMP group. Similar results were seen in the SF groups. However, no differences were noted in the intergroup comparison (CM vs. SF).

For HIF-1α gene expression on EDD 11, slight but non-significant differences were seen, with higher values in the CM control group compared to the CM + iPRF and CM + EMP groups. In the SF intragroup comparison, the SF + iPRF group exhibited higher gene expression levels than other groups, with the SF + iPRF group showing elevated expression compared to the negative control. In the intergroup comparison, the CM + NaCl group displayed higher values than the SF + NaCl group, the SF + iPRF group exhibited higher gene expression levels than the CM + iPRF group.

On EDD 14, the CM + NaCl group exhibited higher expression levels than the other groups. All values were comparable to the negative control group, with no discernible differences noted in the intergroup comparison (CM vs. SF).

For MMP-13 gene expression analysis on EDD 11, higher expression levels were observed in both the CM and SF groups treated with iPRF compared to the matrices rehydrated with NaCl, those biofunctionalized with EMP, and the negative control group. Slightly higher expression values were detected in the intergroup comparison for the CM + NaCl group. The CM group biofunctionalized with iPRF and EMP showed higher expression levels than their SF counterparts (SF + iPRF and SF + EMP).

On EDD 14, the CM control group and CM + iPRF group exhibited higher expression levels than the CM + EMP group. No significant differences were noted in the intergroup comparison.

No differences were observed in the CM and SF intragroup comparison for NOS2 gene expression analysis neither on EDD 11 nor on EDD 14.

**Table 4:** Gene expression analysis for VEGF, HIF-1ɑ, MMP-13, and NOS2 at EDD 11 and EDD 14 for all groups. Displayed are relative gene expression values as mean ± standard deviation

	EDD	CM + NaCl	CM + iPRF	CM + EMP	SF + NaCl	SF + iPRF	SF + EMP
VEGF	11	3.17 ± 1.12	2.35 ± 1.34	1.39 ± 0.86	2.83 ± 2.38	1.76 ± 1.32	1.42 ± 0.67
	14	2.55 ± 2.00	0.99 ± 0.41	0.85 ± 0.76	1.45 ± 0.51	0.84 ± 0.45	0.75 ± 0.31
HIF-1ɑ	11	0.96 ± 0.72	0.53 ± 0.07	0.45 ± 0.06	0.54 ± 0.38	0.72 ± 0.24	0.38 ± 0.22
	14	0.67 ± 0.67	0.33 ± 0.14	0.40 ± 0.17	0.31 ± 0.07	0.23 ± 0.06	0.60 ± 0.46
MMP-13	11	1.22 ± 1.71	2.12 ± 1.52	0.60 ± 0.54	0.43 ± 0.24	1.30 ± 1.02	0.31 ± 0.27
	14	0.81 ± 0.38	0.94 ± 0.91	0.59 ± 0.31	0.64 ± 0.51	0.54 ± 0.20	0.83 ± 0.58
NOS2	11	5.67 ± 8.57	8.65 ± 12.22	3.07 ± 5.56	7.62 ± 9.11	6.38 ± 6.35	3.00 ± 5.00
	14	0.94 ± 0.54	2.31 ± 2.34	1.90 ± 0.75	2.70 ± 1.74	0.84 ± 0.74	5.57 ± 4.62
Number of probes	11	3	4	5	5	5	5
	14	7	12	9	11	14	13

### Histological staining

**Fig. 9:**
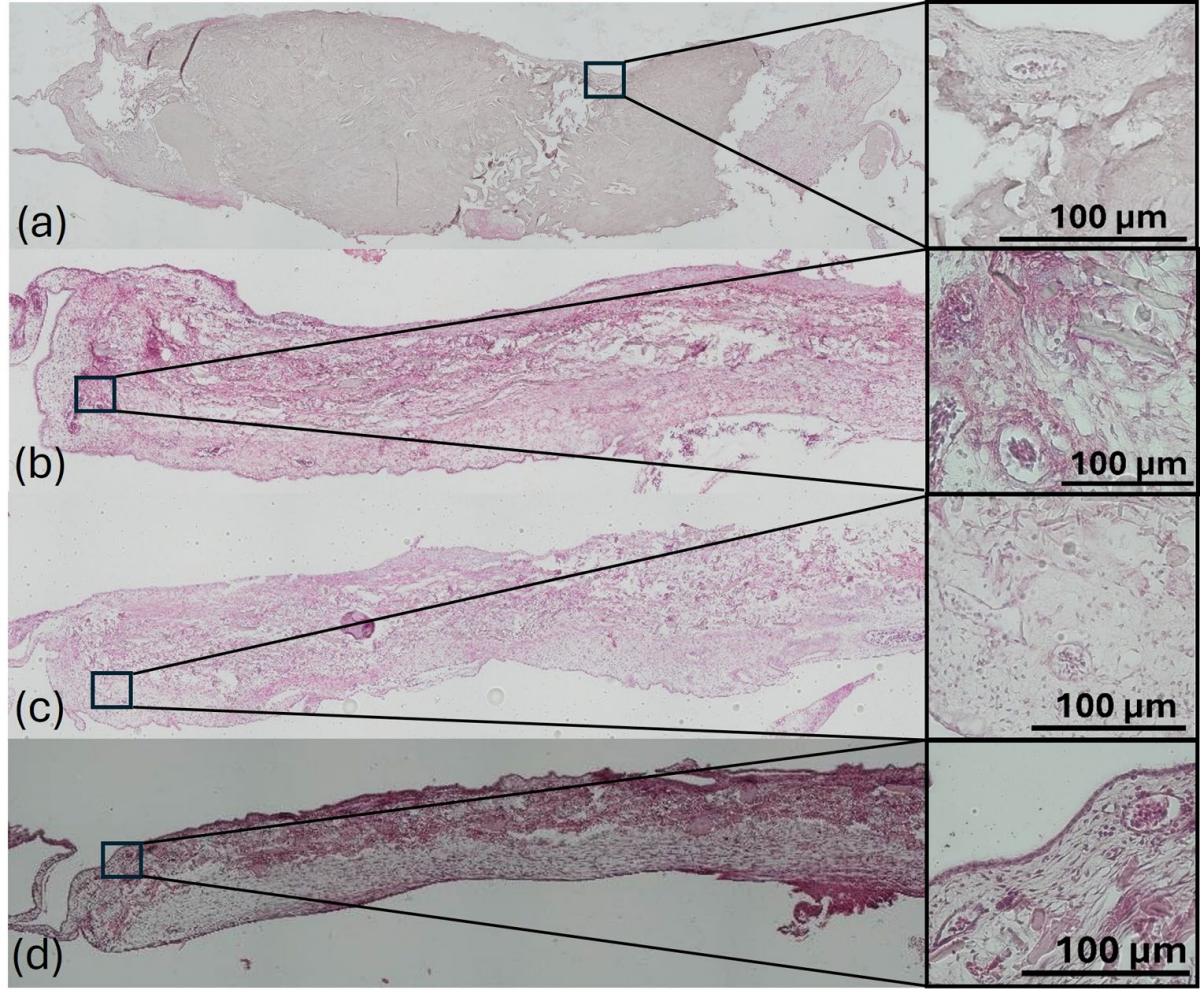
HE-staining of a collagen and silk fibroin matrices. The images were captured at 50x magnification under a light microscope. (**a**) shows a collagen matrix rehydrated with iPRF. In (**b–d**), the silk fibroin matrices of the control group (**b**), the SF + EMP group (**c**), and the SF + iPRF group (**d**) are displayed. The images on the right show the respective enlarged sections, which highlight the vascularized peripheral regions of the matrices in greater detail. (**a–b**) depict the infiltration of connective tissue and cells into the matrices

*A*dditionally histological sections were prepared and HE stained to assess the integration of the matrices into the CAM and the vascularization. The CM and SF matrices differed significantly in their histological structure. In the sections, the CM appeared as a cubic structure with a substantially greater thickness than its silk counterpart. Internally, the CM exhibited a homogeneous appearance. The collagen fibers were intertwined, leaving almost no visible voids between them (Fig. 9). The surface of the CM appeared linear and rigid, with the matrix demarcated from the surrounding CAM tissue. In contrast, the significantly thinner SF matrix gradually transitioned into the CAM tissue. The interior of the matrix appeared heterogeneous, with numerous voids and thin filaments, along with larger, rounded sections of silk fibers that displayed a homogeneous core (Fig. 9). The bends in the matrix surface made the SF matrices appear mechanically less stable and more flexible than the CM.

All matrices that showed attachment to the CAM were entirely surrounded by CAM tissue. In all groups, small clusters of infiltrating cells could be detected in the peripheral margins of the matrices, which, based on morphology, were likely immune cells. However, these were observed sporadically and did not form a lymphocytic ring around the matrix (Fig. 9b). While only a few cells were visible within the CM, they were ubiquitously observed in the SF matrices. The edges showed a tendency for denser cell colonization compared to the central areas of the matrix.

Additionally, small blood vessels were detected in the peripheral areas of the SF matrices and the CM + iPRF group, identifiable by the enclosed erythrocytes (Fig. 9a). Small capillaries were also found at the transitions from the matrix to the CAM tissue. No vessels could be detected within the matrices themselves.

## Discussion

Using the CAM assay, this study tested collagen and silk fibroin matrices for angiogenesis and immunological tolerance. Additionally, the influence of biofunctionalization of both matrices with iPRF and EMP was investigated.

The total number of vascularized specimens was significantly greater in SF matrices, irrespective of the applied treatment. In contrast, within the CM group, only the matrices biofunctionalized with iPRF demonstrated adequate adhesion to the CAM and successful vascularization. Notably, especially compared to the SF + iPRF group, the CM biofunctionalized with iPRF group showed the highest values for the total vascularized area (CM + iPRF: 59.44% vs. SF + iPRF: 34.29%; *p* = 0.031), vessel density (0.0061; *p* < 0.001), and the number of vessel junctions (11.04; *p* = 0.025). However, gene expression data exhibited high variability, with no statistically significant differences between the groups.

In line with this study, Watchararot et al. demonstrated that this material is angiocompatible even on the CAM. It could be ensured that the released degradation products of the matrix do not lead to vessel irritation [15]. After six days, vessel ingrowth was observed without modifying the matrix, which confirms the results by this study [15]. Smeets et al. used native and modified SF-based matrices as a barrier membrane for targeted bone regeneration in vivo. They demonstrated that collagen matrices were significantly faster resorbed than SF-based matrices in vivo, and SF matrices modified with β-TCP contributed to improved new bone formation [11]. Since, in this study, the observation period was rather short compared to the in vivo study by Smeets et al. no such conclusions could be drawn here. Türkkan et al. obtained similar results in vitro using a self-designed SF/PCLPEG-PCL matrix [34]. Luo et al. developed an SF matrix with active co-expression of angiogenesis factors by plasmid transfection. They observed that it contributed to significantly increased angiogenesis compared to the non-modified membranes in the CAM assay. The subsequent animal experiment showed significantly faster dermal wound healing when transfected membranes were placed under the split skin graft [35]. Since most studies did not analyze the effect of iPRF or EMP on angiogenesis and wound healing direct comparisons between the studies are difficult. Watchararot et al. seeded the SF matrix with stem cells. They thereby achieved a significantly increased pro-angiogenic effect [15], which could be confirmed *in ovo* by the experiments of Woloszyk et al. [36–38]. In conclusion, angiocompatibility of natives SF matrices has been shown by several studies confirming the observations during this study. Furthermore, several modifications of SF matrices have been tested, in vitro, *in ovo* and in vivo leading to improved wound healing and angiogenesis as has been shown by this study as well.

In this study, histological analysis revealed marginal vessel infiltration into both matrices. However, notably higher proportions of native matrices (rehydration with NaCl) exhibited vascularization and adherence to the CAM in the SF group at EDD 14 (100% vs. 0%). In this group, 35.79% of the SF matrices demonstrated vessel overgrowth or ingrowth, with the highest average increase in vascularization between EDD 12 and 14. Due to the lack of vascularization in the CM control group, meaningful comparisons are not feasible. These findings align with prior studies indicating the angiocompatibility of SF and its promotion of vessel ingrowth in the CAM assay [15].

Biofunctionalization with either iPRF or EMP yielded markedly distinct outcomes. In CM, iPRF treatment resulted in an early vascularization of the matrix, with 93.33% of specimens exhibiting vessel overgrowth or ingrowth by EDD 12. Conversely, neither the control nor the EMP-treated group showed any signs of vascularization. In contrast, the SF group biofunctionalized with iPRF demonstrated rapid initial vessel ingrowth at EDD 12 (53.33%), albeit with lower values than the other groups two days later (80% vs. 100% and 92.86%). Across all SF groups, the increase in the vascularized area of the matrix was high. In the SF control group, the average vascularized area increased from 14.4 to 35.79%, in the SF + iPRF group from 28.65 to 34.39%, and in the SF + EMP group from 30.97 to 39.12%. While the SF control group showed the highest increase in the vascularized area from EDD 12 to EDD14, the initial vascularization of the iPRF and the EMP group was already high. However, neither group exhibited values above 40%. Consistent with the number of vascularized matrices at EDD 12, the iPRF groups exhibited the highest vessel density and junctions. Two days later, vessel density and the number of vessel junctions were highest in the CM + iPRF group, whereas SF matrices treated with iPRF even showed a decrease in vessel density and only a moderate increase in the number of vessel junctions, which might be attributed to a more pronounced enlargement of the vessels rather than an increase in their number. In the SF + EMP group, vessel density was low on EDD 12 rising to even higher values compared to the group treated with iPRF. A similar trend was observed for the number of vessel junctions, suggesting a pronounced proangiogenic effect compared to iPRF.

Other studies have also corroborated the positive influence of collagen matrices (such as mucoderm^®^, jason^®^, collprotect^®^) rehydrated with PRF on angiogenesis in the CAM assay [26]. Comparable data for neovascularizing silk matrices biofunctionalized with PRF are not yet available. In vitro, Blatt et al. demonstrated that growth factor release (VEGF, TGF-β) was highest 24 h after the application of PRF, which could explain the initially higher vascularization in SF matrices. In contrast to the results of this study, the authors observed a significant increase in the angiogenic potential after 24 h in the *in ovo* yolk sac membrane (YSM) assay. This difference might be attributed to the choice of the assay (YSM vs. CAM), the earlier application of the collagen matrices at EDD 5, and the use of a PRF clot instead of liquid iPRF in this study [26].

Particularly, the earlier developmental stage of the egg in their study might enhance responsiveness to PRF. In their study, Blatt et al. further analyzed the effect of PRF on CM in the CAM assay. Matrix application was performed at EDD 11, and 24 h later, FITC dextran was injected for intravital fluorescence microscopy. Analogously to our study, the authors demonstrated more vessels and branching points in the groups treated with PRF [26]. Using the CAM assay, Schröger et al. conducted a similar assessment of the effect of either liquid or solid PRF on the vascularization of a new porcine-derived collagen membrane (NovoMatrix^®^). Consistent with our study, vessel area, length, thickness, and branching points increased over time, starting at relatively low values (lower than native CM). The authors attributed this effect to the acidity of iPRF compared to saline solution [39]. Interestingly, solid PRF appears not to lower the pH during a 20-minute observation interval when applied to CM, thus exhibiting different effects on wound healing and angiogenesis than liquid PRF [40]. In another study, Blatt et al. analyzed the effect of solid PRF on differences in the vascularization of either porcine or bovine CM. Similar to our study, the porcine CM (BioGide^®^) treated with PRF initially showed fewer vessels than the native group. After a 48-hour interval, the number of vessels per mm^2^ increased significantly. Later, the total number of vessels measured using the YSM assay was similar in both groups. Contradictory findings were observed for bovine CM (Symbios^®^), starting with more vessels in the PRF groups, which decreased after 48 h and increased again later [40].

Similar to the observations made by Vokurka et al., the addition of EMP to CM did not show any positive effect on vascularization in our study. Using a rabbit model, the authors combined PRF, PRP (platelet-rich plasma), or EMP with a bilayered CM for soft tissue augmentation. After a healing period of 1, 7, and 28 days, only the EMP group showed slower angiogenesis and a higher presence of inflammatory infiltrate [30].

Gene expression analysis via qPCR was performed to assess the impact of the different matrices and treatment methods on proangiogenic factors. Though VEGF is one of the most potent endothelial-specific mitogen-promoting angiogenesis, neither EDD 11 nor EDD 14 qPCR analyses showed any relevant statistically significant difference for gene expression of VEGF in either group. Interestingly, values of the matrices treated with NaCl did show the highest gene expression. In line with the natural progression of CAM tissue development, VEGF gene expression was higher on EDD 11 than on EDD 14. The CAM displays a naturally high mitotic rate until EDD 10, after which the mitotic rate gradually declines. Consequently, there is a natural increase in vascularization during the initial ten days of development [41]. During this period, evaluating the effects of treatments aimed at stimulating angiogenesis is challenging due to the substantial amount of neovascularization occurring naturally. Therefore, later time points from EDD 10 onwards were deliberately chosen for measurements of the angiogenic response to enable assessment of neovascularization under more stable conditions following the highly vasoproliferative phase of CAM tissue.

HIF-1ɑ is known for its pro-angiogenetic and cell proliferation-promoting effects [42]. The HIF-1ɑ values at EDD 11 in any group did not show any statistically significant differences from the control.

In addition to vascularization, immune response to native and biofunctionalized matrices should be assessed. Certainly, comparisons between immune responses in adult mammals and those in avian embryonic systems must be made cautiously. However, observations have indicated the presence of macrophage-like cells shortly after the onset of egg development. These cells have demonstrated phagocytic activity, suggesting similarities to mammalian fetal macrophages [43]. From EDD 10 to EDD 14, two primary types of immune cells are recognized in chicken embryos. Firstly, heterophils are predominant, considered the avian equivalent of mammalian neutrophils. Secondly, immune cells belonging to the monocyte/macrophage lineage are identified. These cells are the main producers of MMP-13 in chicken embryos [44–46]. Matrix metalloproteinases (MMPs) are extracellular zinc-dependent endopeptidases involved in the degradation and remodeling of the extracellular matrix in various physiological and pathological processes. Several MMPs, including MMP-13, are known to play crucial roles in neovascularization. The new blood vessel formation process relies on the proteolytic activities facilitated by MMPs [47–49]. In this regard, heterophils and macrophages are likely to have significant roles as sources of these MMPs at sites of tissue remodeling and neovascularization surrounding the transplant. MMP-13 has been linked to collagen remodeling and the initiation of angiogenesis in the CAM assay. Studies have demonstrated its rapid induction in response to angiogenic stimuli, coinciding with early events in vessel formation [44].

Consequently, MMP-13 appears to play a role in initiating angiogenesis. This notion is further supported by evidence showing that MMP-13 can stimulate the secretion of VEGF from endothelial cells and fibroblasts [49]. Interestingly, only in iPRF-treated groups did MMP-13 gene expression show a typical pattern of high values at EDD 11, especially in the CM group, decreasing to lower values at EDD 14. In all other groups, both control and EMP-treated, MMP-13 gene expression was lower in the earlier days of egg development. This might be attributed to a reaction to the foreign blood components of the human donor. Since few studies analyze the effect of platelet concentrates on MMP-13 gene expression, interpretations are difficult. Lo Monaco et al. measured the effect of L-PRF on articular chondrocytes. They showed that L-PRF stimulated the expression of MMP-13 [50].

NOS2 is a cell expression marker for M1 macrophages in mammalian cells, indicating pro-inflammatory activity within our model. While the specific immunological processes in avian species remain largely unexplored, similarities to murine and human models are hypothesized, particularly regarding increased NOS2 activity following macrophage interaction with pathogens [43]. Interestingly, gene expression was comparably high only in the SF + iPRF group on EDD 11. In line with the results of the MMP-13 gene expression analysis, this phenomenon could be attributed to an inflammatory reaction to the foreign blood concentrate (xenogeneic material from human to chicken) [30] and the acidity of iPRF compared to saline solution as shown by Schröger et al. [39].

Nevertheless, biofunctionalization with iPRF did show a positive effect on clinically observable vascularization and no difference in histological inflammation compared to the other groups, indicating a less harmful and more advantageous action of this mechanism.

The observed high variances in gene expression analysis appear to stem from various factors that could not all be fully addressed within the confines of this study. Firstly, it is important to note that the samples for qPCR were obtained from living animals, resulting in inherent inter-individual variability. Our study covered a specific time period rather than a single point, posing a unique challenge for qPCR analysis. Finding a stable reference gene throughout embryonic development, characterized by significant changes in gene expression within days, is challenging. Additionally, egg development is not synchronized beyond the initial breeding, leading to variations in developmental pace among eggs, which may manifest visually and likely influence gene expression profiles. Despite efforts to optimize the qPCR methodology, including duplicate analyses of the entire sample set, certain limitations persist. Unfortunately, the qPCR analysis encountered various challenges due to differences among the genes studied. Finding primers suitable for the chicken transcriptome posed difficulties, and gene expression levels for certain genes on specific days approached the detection limit. Additionally, RNA isolation failed in some samples, and certain melting curves indicated nonspecific primer binding, necessitating their exclusion from the analysis.

Another complication arose from the limited research on the avian embryonic immune system, as details about the types of cells present during different developmental stages remain unknown. Furthermore, there is a lack of knowledge regarding the cellular markers expressed by these cells. The immune system of the CAM begins to develop from EDD 11 [51] and is only partially active until EDD 15 [52]. Therefore, making statements about the immunological tolerance of xenogeneic materials tested on the CAM is limited.

Another disadvantage of the CAM assay is the high mortality rate of the embryos. This study excluded 30 to 60% of chicken embryos from the experimental evaluation depending on the experimental run. Particularly high mortality occurred one day after the application of matrices with iPRF. This could be due to human blood antibodies, which may have led to immunological reactions in the chicken embryo. However, there are currently no research results supporting this hypothesis.

In this study, utilizing the CAM assay, we demonstrated a relatively low angiogenic potential of native collagen membranes which the addition of iPRF could significantly enhance. Particularly in thicker collagen membranes, the 26-fold higher storage of iPRF resulted in an early induction of MMP-13, explaining the initially lower vascularization rate and the substantial increase after seven days. Enamel matrix proteins caused a reduction in vascularization, especially in collagen membranes, which likely retained more active substances than the thinner silk-fibroin matrix. Regardless of the rehydration process, silk fibroin matrices exhibited a high vascularization rate. However, the vascularized surface area, encompassing the number of vessel junctions and vessel density, was lower than that of collagen membranes treated with iPRF.

## Conclusions

Biofunctionalization with iPRF in matrices capable of retaining higher volumes of liquid substances leads to a high vascularization rate, suggesting improved intraoral wound healing after guided tissue regeneration (GTR). Moreover, despite biofunctionalization, very thin SF matrices exhibit a high vascularization capacity, indicating SF as a promising material for intraoral soft tissue reconstruction. Further investigation into the effect of biofunctionalization on SF matrices should explore additional substances and matrices of varying thicknesses and compositions.

## Data Availability

The datasets used and/or analysed during the current study are available from the corresponding author on reasonable request.

## Abbreviations

CAM: Chorioallantoic membrane
CM: Collagen matrix
EDD: Egg development day
EMP: Enamel matrix protein
GTR: Guided tissue regeneration
HE: Hematoxylin eosin
HIF-1ɑ: Hypoxia inducible factor-1ɑ
iPRF: Injectable platelet-rich-fibrin
MMP-13: Matrix metallopeptidase 13
NaCl: Sodium chloride
NOS2: Nitric oxide synthase 2
PRP: Platelet-rich plasma
qPCR: Quantitative polymerase chain reaction
SF: Silk fibroin
VEGF: Vascular endothelial growth factor
YSM: Yolk sac membrane
